# Tolfenamic acid inhibits GSK-3β and PP2A mediated tau hyperphosphorylation in Alzheimer’s disease models

**DOI:** 10.1186/s12576-020-00757-y

**Published:** 2020-06-09

**Authors:** Huiming Zhang, Xiaojuan Wang, Pu Xu, Xuefei Ji, Tianyan Chi, Peng Liu, Libo Zou

**Affiliations:** Department of Pharmacology, Shenyang Pharmaceutical University, 103 Wenhua Road, Shenhe District, Shenyang, 110016 Liaoning People’s Republic of China

**Keywords:** Alzheimer’s disease, Glycogen synthase kinase-3β, Protein phosphatase 2A, Tau hyperphosphorylation, Tolfenamic acid

## Abstract

Tolfenamic acid, a nonsteroidal anti-inflammatory drug, alleviated learning and memory deficits and decreased the expression of specificity protein 1 (SP1)-mediated cyclin-dependent kinase-5 (CDK5), a major protein kinase that regulates hyperphosphorylated tau, in Alzheimer’s disease (AD) transgenic mice. However, whether tolfenamic acid can regulate the major tau protein kinase, glycogen synthase kinase-3β (GSK-3β), or tau protein phosphatase, protein phosphatase 2A (PP2A), further inhibiting hyperphosphorylation of tau, remains unknown. To this end, tolfenamic acid was administered i.p. in a GSK-3β overactivation postnatal rat model and orally in mice after intracerebroventricular (ICV) injection of okadaic acid (OA) to develop a PP2A inhibition model. We used four behavioural experiments to evaluate memory function in ICV-OA mice. In this study, tolfenamic acid attenuated memory dysfunction. Tolfenamic acid decreased the expression of hyperphosphorylated tau in the brain by inhibiting GSK-3β activity, decreasing phosphorylated PP2A (Tyr307), and enhancing PP2A activity. Tolfenamic acid also increased wortmannin (WT) and GF-109203X (GFX) induced phosphorylation of GSK-3β (Ser9) and prevented OA-induced downregulation of PP2A activity in PC12 cells. Altogether, these results show that tolfenamic acid not only decreased SP1/CDK5-mediated tau phosphorylation, but also inhibited GSK-3β and PP2A-mediated tau hyperphosphorylation in AD models.

## Introduction

The World Alzheimer Report in 2018 showed that the number of people with dementia worldwide is currently 50 million, but will reach 152 million in 2050 [1]. Alzheimer’s disease (AD) is a global threat to human health [2–4]. The clinical hallmarks of AD are cognitive and psychiatric dysfunction. The major neuropathological hallmarks of AD are senile plaque deposition, neurofibrillary tangle formation, and neuronal cell loss [2]. The tau protein, a microtubule-associated protein that stabilizes the neuronal cytoskeleton, can be phosphorylated at specific sites [5]. However, high levels of phosphorylated tau, which destabilizes the neuronal cytoskeleton, have been found in the brains of patients with neurodegenerative disorders [6, 7]. Hyperphosphorylated tau causes mitochondrial dysfunction, synapse impairment, and cognitive disorders [8].

Specificity protein 1 (Sp1) is a transcription factor, and its target genes include amyloid precursor protein (APP), beta-site amyloid precursor protein-cleaving enzyme 1 (BACE1), and CDK5, all of which play vital roles in AD. Therefore, the regulation of SP1 has an important role in AD therapeutic strategies.

Tolfenamic acid (TA) is an SP1 inhibitor [9]. An experimental study of its safety showed that repeated oral administration of 50 mg/kg tolfenamic acid for 6 weeks did not affect mouse body weight and had no toxic effect on mouse organs [10]. Tolfenamic acid attenuated learning and memory deficits in AD transgenic mice through inhibiting the expression of APP, BACE1, and SP1-mediated CDK5/hyperphosphorylated tau [11, 12]. However, whether tolfenamic acid can prevent other protein kinases or protein phosphatases from regulating tau hyperphosphorylation is still unknown.

In this study, we administered tolfenamic acid i.p. in a GSK-3β overactivation postnatal rat model and orally in ICV okadaic acid (OA)-induced mice, a model of PP2A inhibition, to evaluate the effect of tolfenamic acid on hyperphosphorylated tau. The Y-maze test, novel object recognition test, Morris water maze test, and passive avoidance test were used to test the effect of tolfenamic acid on cognitive function in ICV-OA model mice. Furthermore, we treated PC12 cells with wortmannin (WT, a specific inhibitor of PI3K)/GF-109203X (GFX, a specific inhibitor of PKC) and OA to induce tau hyperphosphorylation and confirm the effects of tolfenamic acid on neurotoxicity and tau phosphorylation and the mechanism of tolfenamic acid.

## Materials and methods

### Materials

Tolfenamic acid (purity ≥ 98%) and antibodies against p-Tau Ser202, p-Tau Ser396, GSK-3β, p-GSK-3β Ser9, and PP2A were purchased from Abcam (MA, USA). Okadaic acid was purchased from Beyotime Biotechnology (Shanghai, China). Wortmannin and GF-109203X were purchased from Selleck Chemicals (Shanghai, China). Antibodies against Akt, p-Akt Ser473, and β-actin, and secondary antibodies were purchased from Proteintech (Wuhan, China). Antibody against p-PP2A Tyr307 was purchased from Sigma-Aldrich (USA).

### Animals

Two-month-old male and female Wistar rats and male C57BL/6 J mice (22 to 25 g) were purchased from Liao Ning Chang Sheng Biotechnology Co., Ltd. (Benxi, China). Rat pups at postnatal day 12 (P12) were used for studies of the efficacy of GSK3β inhibition. All animals were maintained in polyacrylic cages under standard housing conditions with a 12-h light/dark cycle. Food and water were provided ad libitum.

### ICV injection

ICV injection was performed as described in our previous report [13]. Briefly, the mice were anaesthetized with 2.5% avertin (Sigma-Aldrich, USA). Then, the head of each mouse was held in a stereotaxis instrument. OA (100 ng in 2 μl of normal saline) or vehicle (2 μl of normal saline) was injected into the right ventricle at the following coordinates: − 0.5 mm anterior to bregma, 1.0 mm lateral, and − 3.0 mm ventral. The OA dose was based on a study by Rajasekar et al. [14].

### Treatment schedule

Experiment A: tolfenamic acid (50 mg/kg) was dissolved in 5% DMSO and injected i.p. in P12 rats from the same litter. At 4 and 6 h after injection, the rats were killed, and the brain tissue was dissected.

**Fig. 1:**
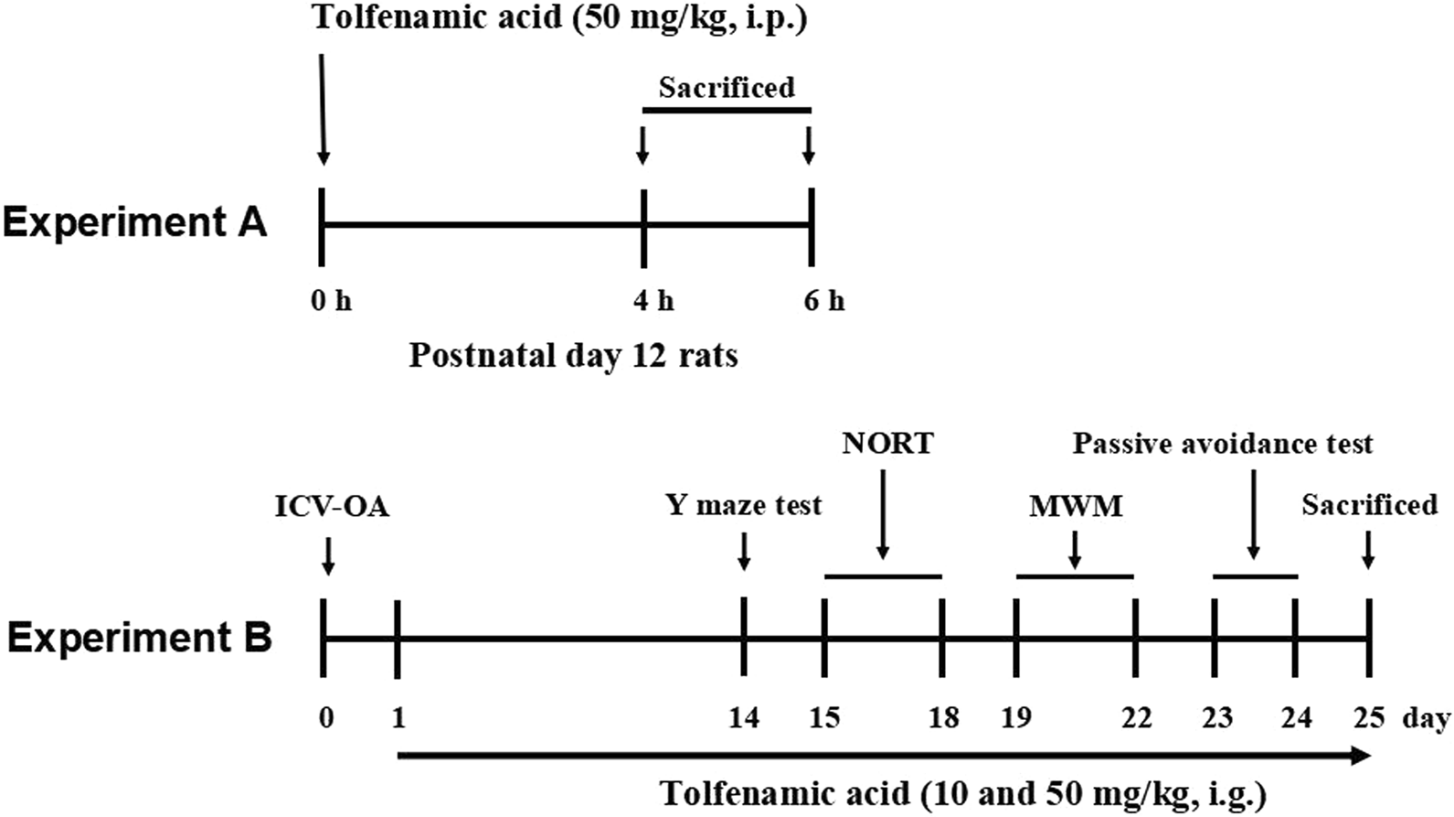
Experimental protocol. Experiment A: treatment with tolfenamic acid on P12 rats for 4 h and 6 h; Experiment B: treatment with tolfenamic acid for 14 days, then perform the behavioral experiments

Experiment B: C57BL/6 J mice were divided into a control group, model group (ICV-OA), a group treated with 10 mg/kg TA and a group treated with 50 mg/kg TA (*n* = 10/group). The mice were given tolfenamic acid or vehicle i.g. After behavioural assessment, the mice were killed, and the brain tissue was dissected. The brains from five mice in each group were used for immunohistochemical staining. The cerebral cortices from the other mice were used for ELISA and a PP2A activity assay. The hippocampi were used for western blotting. Tolfenamic acid doses were selected based on those reported by Adwan et al. [12] and those in our previous study [15]. The experimental schedule is summarized in Fig. 1.

### Y-maze test

The Y-maze test apparatus contained three arms (40 cm long × 10 cm wide × 12 cm high). Each mouse was paced in one arm and allowed to explore for 5 min. The number of entries into each arm and the sequence of these entries were recorded. Entrance of the mouse into the three arms in sequence was referred to as a “successful alternation”. Alternation behaviour was calculated as [number of successful alternations/(total number of arm entries-2) × 100%] [15].

### Novel object recognition test

A square box (50 cm long × 50 cm wide × 15 cm high) was used as equipment for the novel object recognition test. On the first 2 days, the mice were habituated to the equipment 2 times/day for 10 min each. On the test day, the mice were allowed to explore two identical objects, A1 and A2, for 5 min. After a 1 h intertrial interval, A2 was replaced with novel object B, and the mice were returned to the box for 5 min. After a 24-h retention interval, B was replaced with novel object C, and the mice were returned to the box for another 5 min. The exploration time for each object was recorded. The preferential index (PI) was calculated as [time spent exploring a novel object/total exploration time × 100%] [15].

### Morris water maze test

A circular pool (100 cm in diameter, 50 cm high) containing 30-cm-deep water at 25 ± 1 °C was used as the equipment for the Morris water maze test. The fourth quadrant contained a security platform that was 1 cm below the water level. The mice were trained twice a day for 3 days after a 3-h intertrial interval. The mice were allowed to swim for 60 s to find the platform. If the mice failed to find the platform in 60 s, they were placed on the platform for 20 s. On the fourth day, the spatial memory of the mice was tested. The platform was removed, and each mouse was allowed to swim for 60 s. The escape latency (time spent in the fourth quadrant), swimming distance, and swimming speed were recorded [16].

### Passive avoidance test

The equipment used for the passive avoidance test consisted of a bright room and a dark room. A bright light bulb was hanging on the top of the bright room to encourage the mice to enter the dark room, and copper bars at the bottom of the dark room could conduct electricity at a voltage of 31 V. The experiment was carried out on 2 days. On the first day (the training phase), the mice were placed individually in the bright room without electricity and moved freely for 5 min. Then, the mice were driven into the dark room through the use of alternating current. The normal reaction of the mice was to run back to the bright room to avoid electric conduction. Some of the mice repeatedly ran into the dark room, where they were shocked, and then quickly ran back into the bright room. The same method used on the training day was used on the test day after a 24-h retention interval; however, the dark room was not electrified. The number of mice that entered the dark room within 5 min was recorded as the error time [15].

### Immunohistochemical staining

Briefly, brain sections were incubated with antibodies against p-Tau S396 (1:1000) and p-PP2A (1:200) at 4 °C overnight. Then, the sections were incubated with biotin-labelled secondary antibody at 37 °C for 30 min. The sections were treated with avidin–biotin enzyme reagent and visualized using a DAB kit (Boster, Wuhan, China). The positive area in each section was quantified by using ImageJ software [15].

### Western blotting

Protein samples (30 μg) were electrophoresed on 8 to 12% gradient SDS-PAGE gels and transferred to PVDF membranes. The PDVF membranes were then blocked with 5% skim milk for 2 h at 37 °C; incubated with primary antibodies against p-Tau Ser396 (1:1000), p-Tau Ser202 (1:1000), total Tau (1:2000), GSK-3β (1:1000), p-GSK-3β Ser9 (1:2000), PP2A (1:1000), p-PP2A (1:1000), Akt (1:1000), p-Akt (1:800), and β-actin (1:2000) at 4 °C overnight; and finally incubated with secondary antibody for 2 h at room temperature. Protein bands were visualized using an ECL kit. Band intensities were quantified using ImageJ software [15].

### Measurement of PP2A activity

PP2A activity was measured with a kit according to the manufacturer’s protocol (V2460, Promega, USA). Briefly, extracts of cerebral cortex tissue were centrifuged to remove particulate matter, and endogenous free phosphate was then removed with gel chromatography. Samples were incubated with a chemically synthesized phosphopeptide in a 96-well plate at 37 °C for 30 min. The reaction was stopped by the addition of 50 μl of a molybdate dye/additive mixture. The absorbance at 630 nm was measured using a fluorescence reader (Thermo Fisher Scientific, USA).

### Cell viability

PC12 cells were obtained from the National Infrastructure of Cell Line Resource (Beijing, China). The cells were cultured in RPMI-1640 medium containing 10% foetal bovine serum in a cell incubator (5% CO_2_ and 37 °C). The viability of the cells was tested by MTT assay. PC12 cells (1 × 10^4^ cells per well) were plated in 96-well plates. After 24 h of culture, the cells were incubated with tolfenamic acid (5 and 10 μM) for 24 h. Subsequently, the cells were incubated with OA (20 nM) for 6 h or WT (10 μM)/GFX (10 μM) for 3 h. MTT (5 mg/ml) was added to each well. After 4 h, the medium was removed, and 150 μl of DMSO was added to each well. The absorbance at 540 nm was measured using an ELISA reader.

Tolfenamic acid/OA/WT/GFX doses and treatment times were selected based on reports by Adwan et al., Yang et al., and Yang et al. [17–19], and our preliminary experiment.

### Cell-based ELISA

Cell-based colorimetric ELISA kits (ImmunoWay, USA) were used for the quantitative detection of phospho-PP2A (Y307) (KA1325C), phospho-Akt (T308) (KA1015C), phospho-GSK-3β (S9) (KA1578C), phospho-GSK-3ɑ/β (Y279/216) (KA1168C), phospho-Tau (T231) (KA1151C), and phospho-Tau (S396) (KA1668C) in PC12 cells. The absorbance at 450 nm was measured. Then, crystal violet cell staining was used to quantify the cells, and the absorbance at 595 nm was measured. The protein levels were normalized to the total cell number (OD at 450 nm/OD at 595 nm) in each well.

### Statistical analysis

The data were analysed using SPSS 21.0. Statistical significance was determined using one-way or two-way ANOVA followed by Fisher’s LSD multiple comparisons test with homogeneity of variance or Dunnett’s T3 test with heterogeneity of variance. Experimental data are represented as the mean ± SEMs. A value of *p* < 0.05 indicates statistical significance.

## Results

### Tolfenamic acid attenuated tau hyperphosphorylation in postnatal rats

**Fig. 2:**
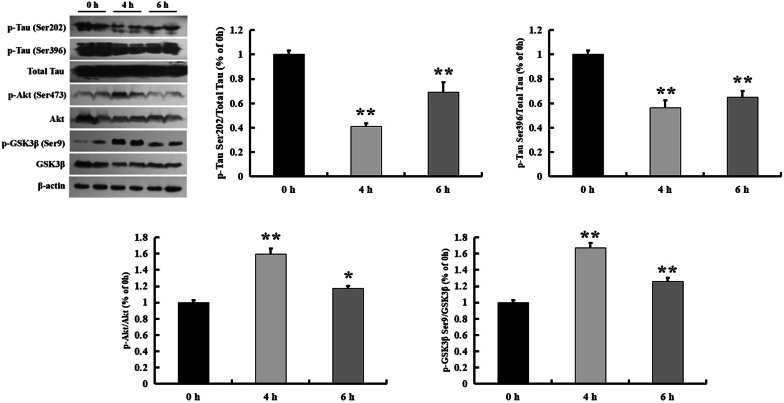
Effect of tolfenamic acid on the expression of p-tau and p-GSK-3β (Ser9) in hippocampus of P12 rats. Tolfenamic acid treatment (50 mg/kg, i.p.) for 4 and 6 h significantly decreased the expression of p-tau (Ser-202 and Ser-396) and increased the expression of p-GSK-3β (Ser9) and p-Akt (Ser473). All results are expressed as the mean ± SEM. *n *= 5; *p < 0.05, **p < 0.01 versus 0 h

As reported by Selenica et al., Takahashi et al., Leroy and Brion, p-tau and p-GSK-3β levels were increased in the developing rat brain and peaked at postnatal day 12 (P12) [20–22]. P12 rats were used for studies of the efficacy of GSK3β inhibition [20]. Tolfenamic acid has been reported to reduce p-tau levels by inhibiting CDK5 expression [12]. In this study, 50 mg/kg tolfenamic acid was injected i.p. for 4 and 6 h. Then, we used western blotting for semi-quantification of the p-tau level in the brain. The p-tau levels were significantly decreased 4 and 6 h after tolfenamic acid treatment (*p* < 0.01, Fig. 2). Tolfenamic acid enhanced p-GSK-3β (Ser9) levels in the brains of the P12 rats and also increased levels of p-Akt (Ser473) (*p* < 0.05, Fig. 2), which is upstream of GSK-3β. We next used a specific cellular model of GSK-3β activation to test the effect of tolfenamic acid on an upstream target of p-tau.

### Tolfenamic acid attenuated tau hyperphosphorylation in WT/GFX-treated PC12 cells

**Fig. 3:**
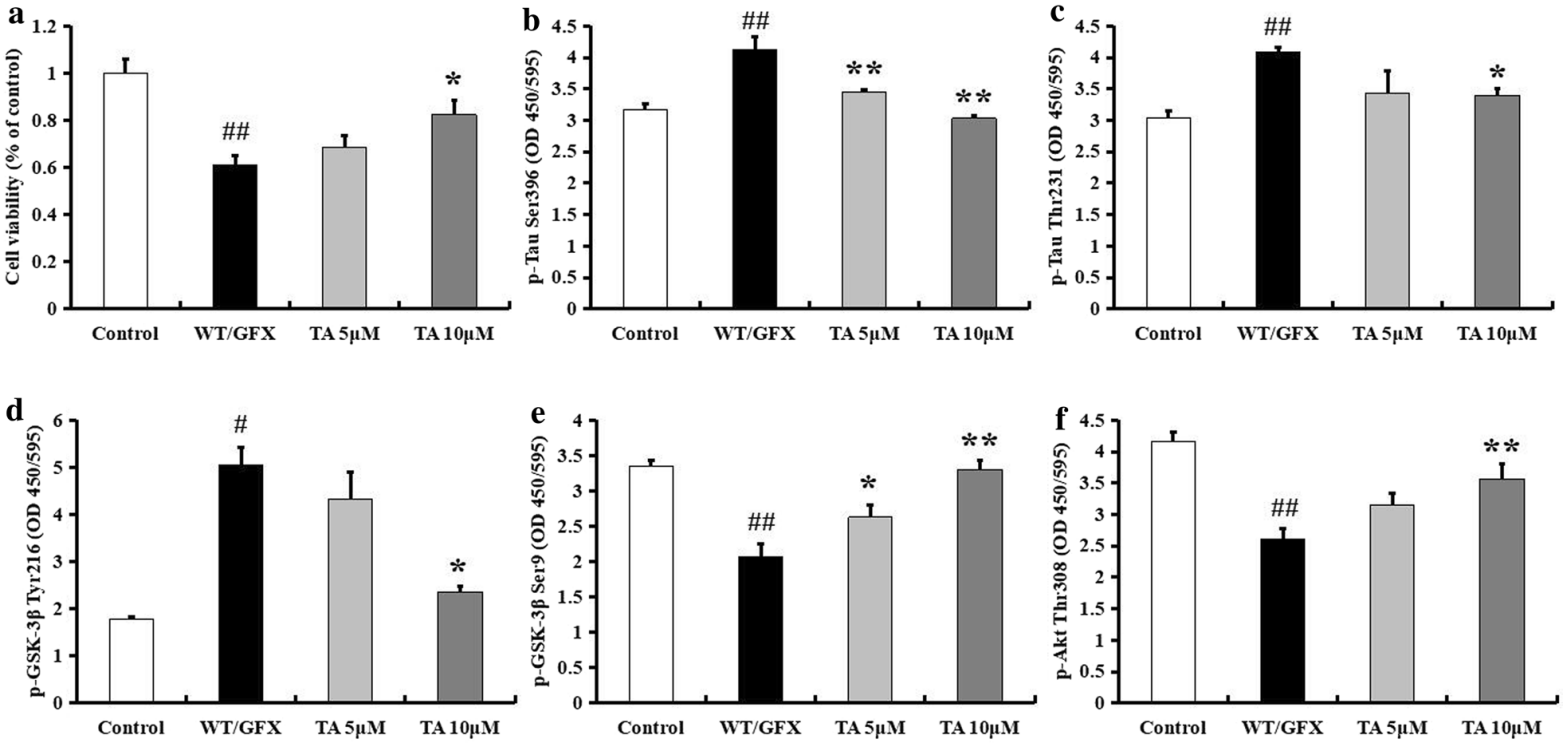
Effect of tolfenamic acid on WT/GFX-treated PC12 cells. PC12 cells were treated with tolfenamic acid (5 and 10 μM) for 24 h, then with WT/GFX for 3 h. Tolfenamic acid treatment significantly prevent WT/GFX-induced cell death (**a**). Tolfenamic acid treatment significantly decreased the expression of p-tau Ser396 (**b**), Thr231 (**c**), p-GSK-3β Tyr216 (**d**) and increased the expression of p-GSK-3β Ser9 (**e**) and p-Akt Thr308 (**f**). All results are expressed as the mean ± SEM. *n* = 3; ^#^*p* < 0.05, ^##^*p* < 0.01 versus control; *p < 0.05, **p < 0.01 versus WT/GFX

The combined use of WT (a PI3K inhibitor) and GFX (a PKC inhibitor) can specifically trigger GSK-3β, further inducing tau hyperphosphorylation. Therefore, we used WT/GFX-treated PC12 cells as an in vitro model to confirm the effect of tolfenamic acid on GSK-3β activation-induced neurotoxicity and tau hyperphosphorylation. Pretreatment with tolfenamic acid for 24 h significantly prevented WT/GFX exposure-induced PC12 cell death (*p* < 0.05, Fig. 3a). Tolfenamic acid also decreased p-tau Ser396, p-tau Thr231, and p-GSK-3β Tyr216 levels (*p* < 0.05, Fig. 3b–d) and enhanced p-GSK-3β Ser9 and p-Akt Thr308 levels (*p* < 0.05, Fig. 3e, f), which was in accordance with the results obtained in postnatal rats. Based on the above in vivo and in vitro results, the inhibitory effect of tolfenamic acid on GSK-3β activity may be one mechanism by which hyperphosphorylation of tau is decreased.

### Tolfenamic acid attenuated cognitive dysfunction induced by ICV-OA in mice

**Fig. 4:**
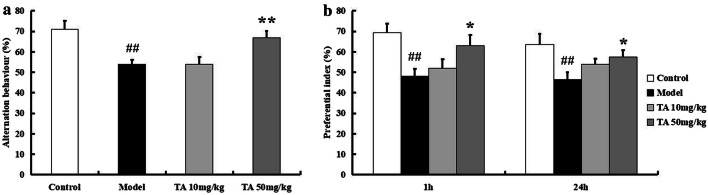
Effect of tolfenamic acid on working memory, memory recall and visual recognition deficits in ICV-OA mice. Model group mice exhibited a decrease in spontaneous alternation behaviour in Y-maze test (**a**), a significant decrease in 1 h and 24 h PI in novel object recognition test (**b**), which compared to control mice. Tolfenamic acid treatment attenuated these cognitive deficits. All results are expressed as the mean ± SEM. *n* = 10; ^##^*p* < 0.01 versus control; *p < 0.05, **p < 0.01 versus model

In addition to protein kinases, protein phosphatases regulate tau phosphorylation and contribute to cognitive dysfunction in AD. OA is an inhibitor of PP2A, the most important protein phosphatase. Injection of OA in the mouse brain can induce cognitive impairments for at least 4 weeks. In this study, we used an ICV-OA mouse model to test the effect of tolfenamic acid on cognitive function and PP2A activity. The Y-maze test was performed to evaluate the working memory of individual mice. Treatment with 50 mg/kg tolfenamic acid alleviated spontaneous alternation behavioural deficits in OA-injected mice (*p* < 0.01, Fig. 4a). The novel object recognition test was performed to evaluate recall memory and visual recognition in individual mice. The PI for novel object B was obviously decreased in the model group (*p* < 0.01, Fig. 4b). Similar results were observed with novel object C. That is, OA injection induced significant memory recall and visual recognition impairments. Tolfenamic acid (50 mg/kg) obviously attenuated this decrease in PI (*p* < 0.05, Fig. 4b).

**Fig. 5:**
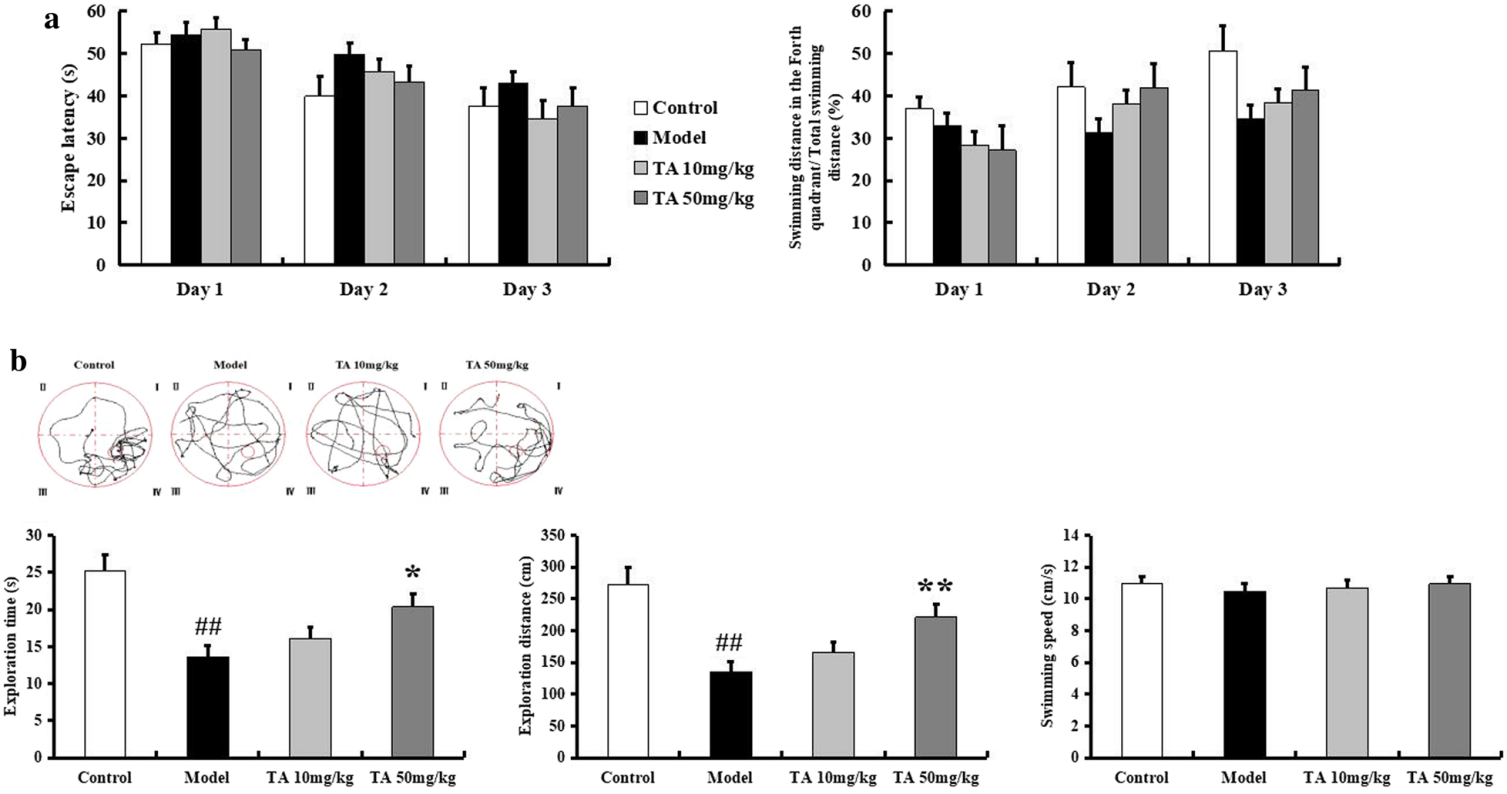
Effect of tolfenamic acid on spatial memory deficits in ICV-OA mice. Model group mice exhibited long swimming time and swimming distance to reach the platform during the training period in Morris water maze test (**a**). Model group mice showed a decrease in the swimming time and swimming distance in the target quadrant in the probe trial (**b**). These effects were reversed by tolfenamic acid. No significant differences in swim speed were observed among the groups (**b**). The results of training period are expressed as the mean ± SEM. The other results are expressed as the mean ± SEM. *n* = 10; ^##^*p* < 0.01 versus control; *p < 0.05, **p < 0.01 versus model

In the Morris water maze test, during the training period, mice in the model group exhibited a longer swimming time and farther swimming distance to find the platform than mice in the control group (Fig. 5a). In the probe test, compared to the control group, the model group exhibited significant decreases in exploration time and exploration distance in the target quadrant (*p* < 0.01, Fig. 5b). Mice in the tolfenamic acid group spent more time and travelled a longer distance in the target quadrant than mice in the model group (*p* < 0.05, Fig. 5b). There were no significant differences in swimming speed among the animal groups (*p* > 0.05, Fig. 5b).

**Fig. 6:**
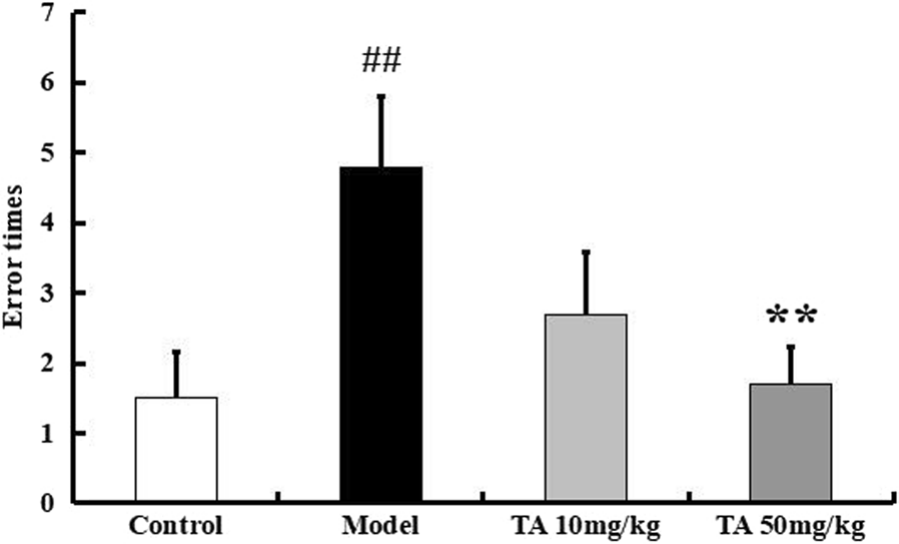
Effect of tolfenamic acid on long-term memory and learning ability in ICV-OA mice. Model group mice exhibited a significant increase in error times, compared to control mice. Tolfenamic acid attenuated the cognitive deficits in passive avoidance test. All results are expressed as the mean ± SEM. *n* = 10; ^##^*p* < 0.01 versus control; **p < 0.01 versus model

The passive avoidance test was performed in the mice to evaluate long-term memory. The error time was obviously increased in the model group compared to the other groups (*p* < 0.01, Fig. 6). The error time in the 50 mg/kg tolfenamic acid group was less than that in the model group (*p* < 0.01, Fig. 6). Thus, tolfenamic acid could attenuate deficits in working memory, spatial memory and long-term memory. We next tested the effect of tolfenamic acid on PP2A inhibition-induced tau phosphorylation.

### Tolfenamic acid attenuated tau hyperphosphorylation in ICV-OA mice

**Fig. 7:**
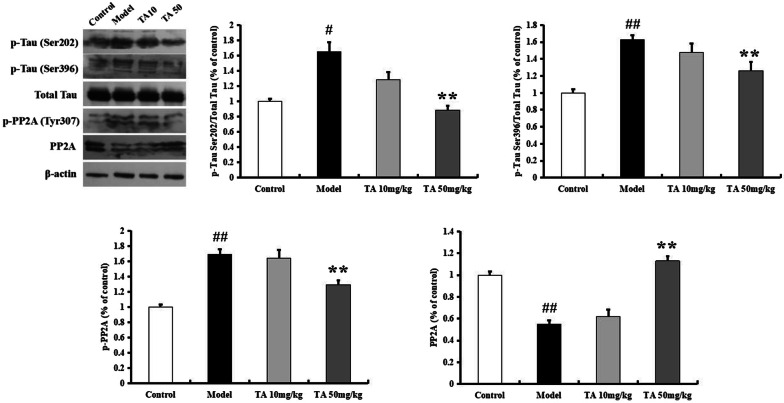
Effect of tolfenamic acid on the expression of p-tau and p-PP2A in hippocampus of ICV-OA mice. Tolfenamic acid treatment significantly decreased the expression of p-tau (Ser202 and Ser396) and p-PP2A (Y307), increased the expression of PP2A. All results are expressed as the mean ± SEM. *n* = 5; ^#^*p* < 0.05, ^##^*p* < 0.01 versus control; *p < 0.05, **p < 0.01 versus model

**Fig. 8:**
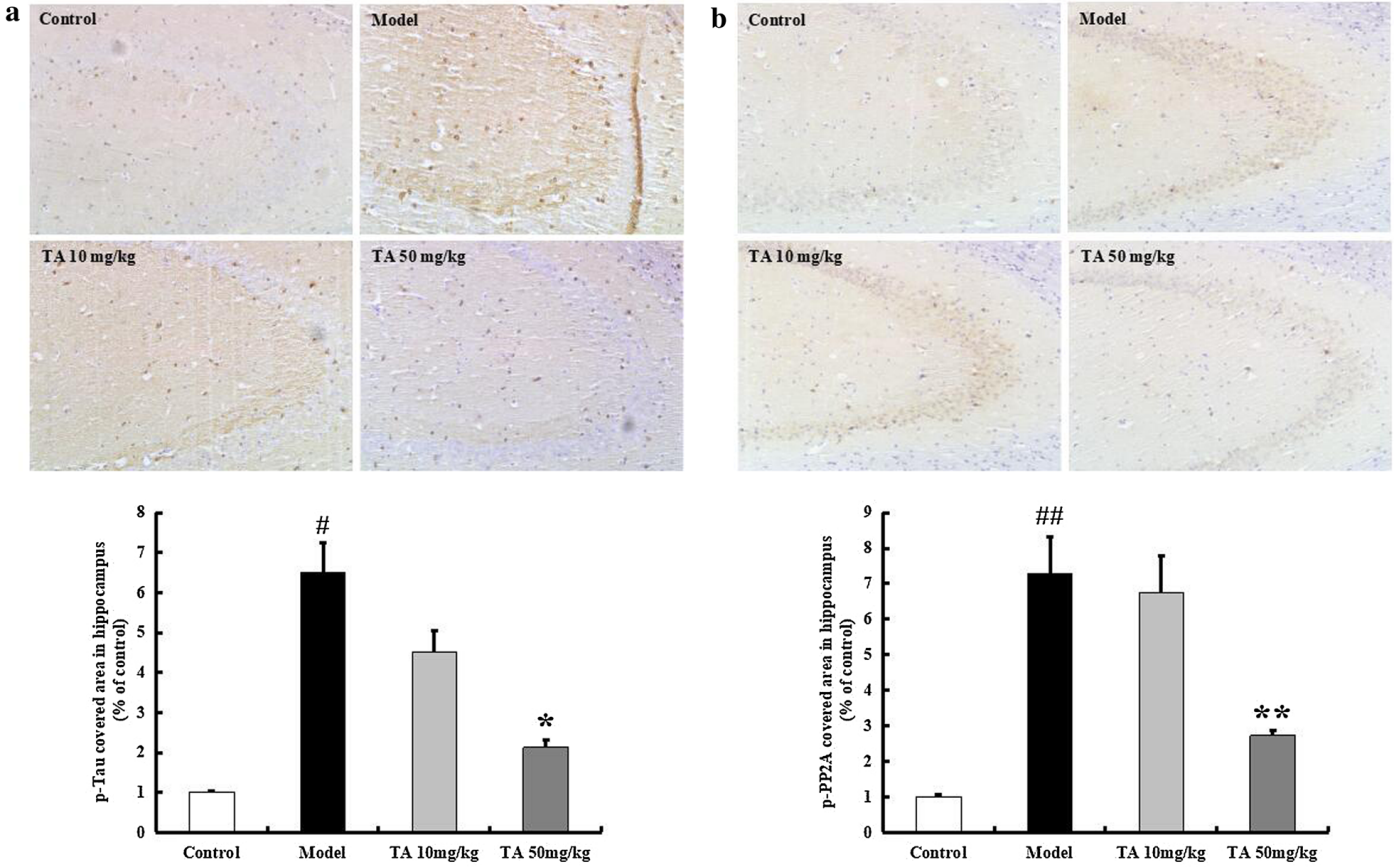
Effect of tolfenamic acid on the expression of p-tau and p-PP2A in hippocampal CA3 region of ICV-OA mice. Tolfenamic acid treatment significantly decreased the expression of p-tau Ser396 (**a**) and p-PP2A Tyr307 (**b**). All results are expressed as the mean ± SEM. n = 4; ^#^*p* < 0.05, ^##^*p* < 0.01 versus control; *p < 0.05, **p < 0.01 versus model

The p-tau level was tested by western blotting and immunohistochemistry and found to be significantly increased in the model group (Ser202, *p* < 0.05; Ser396, *p* < 0.01, Fig. 7). Tolfenamic acid significantly decreased the level of tau phosphorylated at the Ser202 and Ser396 sites (*p* < 0.01, Fig. 7). In the immunohistochemical experiment, the model group mice exhibited high levels of p-tau Ser396 labelling in the hippocampal CA3 region (*p* < 0.05, Fig. 8a). Labelling for p-tau Ser396 was reduced after tolfenamic acid treatment (positive area (%), *p* < 0.05, Fig. 8a).

**Fig. 9:**
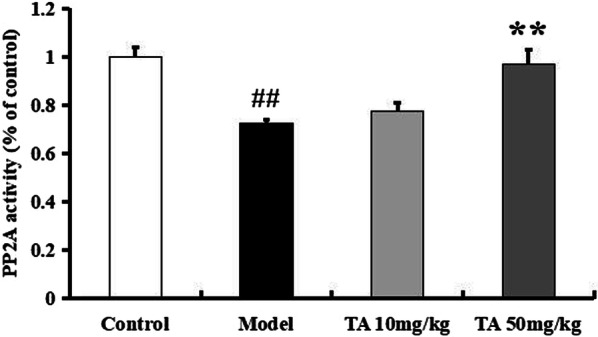
Effect of tolfenamic acid on PP2A activity in cerebral cortex of ICV-OA mice. Tolfenamic acid increased PP2A activity and expression level. All results are expressed as the mean ± SEM. *n* = 4; ^##^*p* < 0.01 versus control; *p < 0.05 versus model

PP2A regulates almost 70% of phosphatase activity, and its phosphorylation inhibits its activity. OA injection increased the expression of p-PP2Ac (Tyr307) (*p *< 0.01, Fig. 7). Tolfenamic acid decreased the p-PP2A level and enhanced the expression of PP2A (*p* < 0.01, Fig. 7). In the immunohistochemical experiment, the model group mice exhibited high levels of p-PP2A labelling in the hippocampal CA3 region, but this increase was prevented by tolfenamic acid (positive area (%), *p* < 0.01, Fig. 8b). We next used a kit to test whether tolfenamic acid enhanced the activity of PP2A. The activity of PP2A in the model group had decreased to 72.65% of that in the control group, but tolfenamic acid prevented this decrease (*p* < 0.01, Fig. 9).

### Tolfenamic acid attenuated tau hyperphosphorylation in OA-treated PC12 cells

**Fig. 10:**
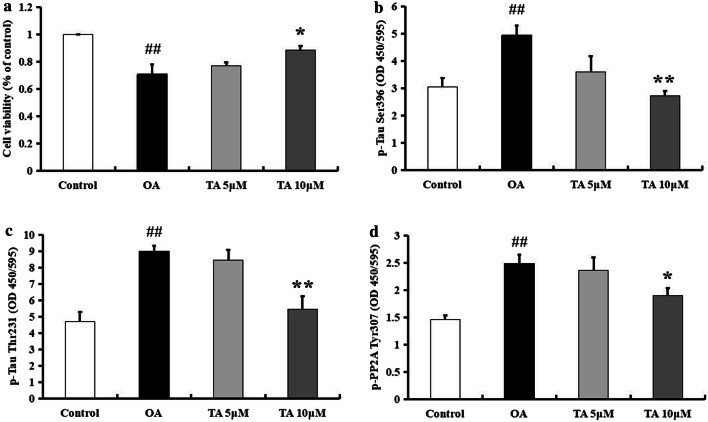
Effect of tolfenamic acid on OA-treated PC12 cells. PC12 cells were treated with tolfenamic acid (5 and 10 μM) for 24 h, then with OA for 6 h. Tolfenamic acid treatment significantly prevent OA-induced cell death (**a**). Tolfenamic acid treatment significantly decreased the expression of p-tau Ser396 (**b**), Thr231 (**c**), and p-PP2A Tyr307 (**d**). All results are expressed as the means ± SEM. *n* = 3; ^##^*p* < 0.01 versus control; *p < 0.05, **p < 0.01 versus OA

We used an in vitro model to confirm the effects of tolfenamic acid on PP2A inhibition-induced neurotoxicity and tau hyperphosphorylation. Pretreatment with tolfenamic acid for 24 h significantly prevented OA exposure-induced PC12 cell death (*p* < 0.01, Fig. 10a). Tolfenamic acid also decreased the expression of p-PP2A (Tyr307) and tau phosphorylated at the Ser396 and Thr231 sites (*p* < 0.05, Fig. 10b–d). The selection of the tolfenamic acid/OA doses and treatment times was based on reports by Adwan et al., Yang et al., and Yang et al. [17–19] and our preliminary experiment. Based on the above in vivo and in vitro results, tolfenamic acid increases PP2A activity, which is a mechanism to prevent hyperphosphorylation of tau and cognitive dysfunction.

## Discussion

Hyperphosphorylated tau aggregates into neurofibrillary tangles (NFTs) in neuronal cells and glial cells in the brain. AD is a tauopathy induced by intraneuronal hyperphosphorylated tau. The literature has reported that NSAIDs can attenuate cognitive deficits in AD animal models and prevent AD pathologies, such as Aβ deposition, the release of neuroinflammatory cytokines and brain cell loss [23–25]. Therefore, some NSAIDs can be considered potential anti-AD agents.

The therapeutic effect of tolfenamic acid on AD animal models was first reported by Professor Zawia and colleagues [26]. Most therapeutic compounds need 2 months or longer to attenuate cognitive dysfunction in AD transgenic mice [11, 27]. However, the use of tolfenamic acid for a very short time (34 days) significantly prevented spatial memory deficits in the Morris water maze test and working memory deficits in the Y-maze test in AD transgenic mice [27, 28]. Tolfenamic acid inhibited the transcription factor Sp1, which reduced the transcription of APP and BACE1 in Pb-exposed human neuroblastoma SH-SY5Y cells and decreased Aβ accumulation in APP transgenic mice [11, 17]. Tolfenamic acid also decreased hyperphosphorylated tau through inhibiting SP1-mediated CDK5 expression in transgenic R1.40 mice with AD [12]. In this study, we report for the first time that tolfenamic acid inhibited tau hyperphosphorylation through inhibiting GSK-3β and activating PP2A.

Protein kinases and phosphatases contribute to tau phosphorylation. As the most important protein kinase, GSK-3β increases p-tau levels. Phosphorylation of GSK-3β at the Ser9 site decreased GSK-3β activity, while phosphorylation of GSK-3β at the Tyr216 site increased GSK-3β activity [29]. Selenica ML et al. reported that both GSK-3β activity and p-tau levels were increased in the brains of developing rats, and this increase peaked at postnatal day 12 (P12 rats) [20]. In addition, GSK-3 inhibitors could reduce tau hyperphosphorylation in P12 model rats, which only required a treatment time of 1 to 4 h [20]. PI3K/Akt is an upstream target that negatively regulates the GSK-3β pathway. Activation of GSK-3β by WT (a PI3K inhibitor) and tau hyperphosphorylation could be reversed by activating PKC [30]. The combined use of WT and GFX (a PKC inhibitor) in the model, which activated GSK-3β, further increased p-tau levels [30]. Therefore, in this study, we used P12 rats and WT/GFX-treated PC12 cells to test the effect of tolfenamic acid on GSK-3β-induced tau hyperphosphorylation. We found that tolfenamic acid decreased the levels of tau phosphorylated at the Ser202, Thr231 and Ser396 sites. Tolfenamic acid also enhanced p-Akt and decreased GSK-3β activity in the brains of P12 rats and in WT/GFX-treated PC12 cells. Thus, tolfenamic acid can decrease hyperphosphorylated tau through inhibiting GSK-3β activity.

The most commonly reported phosphatase in AD is PP2A, a serine/threonine phosphatase. PP2A acts as a suppressor of tau hyperphosphorylation and regulates almost 70% of tau phosphatase activity. Activation of PP2A induces tau dephosphorylation at many sites, including Thr205, Thr212, Thr231, Ser199/202, Ser214, Ser262 and Ser396/404. In the AD patient brain, the activity and protein and mRNA levels of PP-2A were all decreased, and the expression of an endogenous PP2A inhibitor (Inhibitor 2 of Protein Phosphatase 2A, I2PP2A) was increased [31, 32]. OA, a PP2A inhibitor, triggers tau hyperphosphorylation. The literature reports that OA significantly decreased the expression of PP2A in mice, rats, zebrafish and some cell lines in vitro [33–35]. AD-like pathological changes, such as neuronal apoptosis, Ca^2+^ overload, brain energy metabolic abnormalities, and decreases in brain-derived neurotrophic factors, have been found in OA models [32, 36, 37]. ICV-OA for approximately 4 weeks induced cognitive impairments in mice. In our study, tolfenamic acid prevented impairments in working memory, recall memory, visual recognition, spatial memory, and long-term memory. Tolfenamic acid significantly decreased ICV-OA-induced tau hyperphosphorylation at three sites: Ser202, Thr231 and Ser396. By using a PP2A activity detection kit, we found that tolfenamic acid enhanced the activity of PP2A. This may be the mechanism by which tolfenamic acid decreased hyperphosphorylated tau. PP2A activity is decreased by its phosphorylation. The phosphorylation of PP2A at Tyr307 was found to inactivate PP2A and increase abnormally hyperphosphorylated tau, finally causing NFTs [38, 39]. In our study, tolfenamic acid decreased p-PP2A (Tyr307) levels both in vitro and in vivo.

## Conclusions

A previous study showed that tolfenamic acid inhibited protein kinase CDK5-mediated tau hyperphosphorylation in AD transgenic mice [12]. Based on our present study, the list of potential targets by which tolfenamic acid regulates tau phosphorylation has expanded. Tolfenamic acid inhibits the protein kinase GSK-3β and the activity of the protein phosphatase PP2A. As the clinical use of tolfenamic acid is known to be safe, tolfenamic acid may be a candidate for the treatment of AD. Studies have reported that PP2A can regulate GSK-3β through the PI3K/Akt pathway [40, 41]. However, activation of GSK-3β also inhibited PP2A [19]. It is difficult to determine whether GSK-3β is upstream of PP2A or vice versa. In this study, we did not determine whether tolfenamic acid can fit into the binding sites of Akt, GSK-3β or PP2A or directly trigger GSK-3β or PP2A. We cannot rule out that the target of tolfenamic acid is upstream of GSK-3β and PP2A. We will use molecular docking methods, proteomics and systems biology to uncover the molecular mechanisms of tolfenamic acid in our future study.

## Data Availability

The data used to support the findings of this study are available from the corresponding author upon request.

## Abbreviations

AD: Alzheimer’s disease
CDK5: Cyclin-dependent kinase-5
GFX: GF-109203X
GSK-3β: Glycogen synthase kinase-3β
ICV: Intracerebroventricular injection
NFTs: Neurofibrillary tangles
OA: Okadaic acid
PI: Preferential index
PP2A: Protein phosphatase 2A
SP1: Specificity protein 1
TA: Tolfenamic acid
WT: Wortmannin
